# The impact of output price support on smallholder farmers' income: evidence from maize farmers in Ghana

**DOI:** 10.1016/j.heliyon.2020.e05013

**Published:** 2020-09-24

**Authors:** Emmanuel Abokyi, Dirk Strijker, Kofi Fred Asiedu, Michiel N. Daams

**Affiliations:** a Ghana Institute of Management and Public Administration, P. O. Box AH 50, Accra, Ghana; b Department of Economic Geography, Faculty of Spatial Sciences, University of Groningen, P.O.B. 800, Groningen, 9700 AV, the Netherlands; c Department of Cultural Geography, Faculty of Spatial Sciences, University of Groningen, P.O.B. 800, Groningen, 9700 AV, the Netherlands; d KAAF University College, Accra, Ghana; e Nobel International Business School, Accra, Ghana

**Keywords:** Buffer stock, Smallholder farmer, Income, Price support, Coarsened exact matching, Ghana, Food science, Agricultural science, Environmental science, Social sciences, Arts and humanities

## Abstract

Instability in smallholder farmers' income in developing countries due to unstable farm prices has been a challenge for farmers and agricultural policymakers over the years. Sustained price stabilization mechanisms are mostly lacking. In some countries, output price support has been initiated to stabilize incomes and as an incentive to enhance farmer investment and boost production. This paper investigates the impacts of output price support on smallholder farmers' income in Ghana, using a household and farm-level data from 252 beneficiaries and 268 non-beneficiaries of buffer stock operations in Ghana. We applied the Coarsened Exact Matching and Propensity Score Matching methods to balance the data among the two groups. We estimate the smallholder farmer income effect from participating in buffer stock operations by combining the matching methods in a regression framework. The results affirm that buffer stock operations increase the incomes of participating smallholder farming households by at least 12%, providing evidence that output price support via buffer stocks is a critical tool for improving incomes and alleviating poverty among farmers in Ghana. The results further indicate that age, gender, access to market, and use of extension services, as well as transport and packaging costs, drive the participation of smallholder farmers in the buffer stock operations in Ghana. The findings are relevant to local policymakers and development partners who develop tailored interventions to stabilize and increase income for smallholder maize farmers in Ghana.

## 1 Introduction

In Asia and sub-Saharan Africa, incomes of smallholder farmers are low and characterized by sharp fluctuations (Fanzo, 2017). This income instability is due to several factors that influence the supply of agricultural produce, such as weather changes and biological crises like disease outbreaks and pest invasions (Atozou and Lawin, 2016). With agricultural production taking considerable time to complete, the cyclical shortage in supply leads to uncertainty in returns (Gilbert and Morgan, 2010). In turn, volatility in returns leads to risk for farmers, consumers, and other actors in the agriculture value chain, such as finance providers (Demeke and Balié, 2016).

Crucially, inelasticity of supply of agriculture produce may cause an imbalance in agricultural output markets, inducing output price volatility: the rise or fall of producer prices beyond the expectations of consumers and farmers in both international and domestic markets (Abokyi et al., 2018). In most developing countries, output price volatility is made worse by market failures resulting from insecure property rights, inadequate access to the market, the lack of good roads and storage facilities, and incomplete market information (Demeke and Balié, 2016). It must, again, be noted that not all price variations are problematic, as long as price movements follow a well-established pattern in line with market fundamentals. However, this is not the case for most developing countries (Arezki et al., 2016; Demeke and Balié, 2016). Policymakers in developing countries are therefore challenged by the substantial uncertainty regarding the prices of primary commodities.

Among the policy options used in the past by governments in both developed and developing countries to address low and unstable incomes is price support through buffer stock to protect farmers (Deuss, 2015; Pu and Zheng, 2018). For instance, price support via buffer stock operation was a significant component of the Common Agricultural Policy (CAP) in the European Union (Deuss, 2015). Today, price support via minimum price procurement to protect the interest of producers is an integral part of, for example, the Chinese agricultural marketing system (Pu and Zheng, 2018). In Ghana, the government has implemented a price support policy via buffer stock operations for a decade to protect smallholder farmers' income. This study analyses the impact of this price support via buffer stock policy on smallholder farmers' income.

Low and unstable farm income, especially for smallholders, has continuously rationalized public support to farmers for welfare improvement in many developed countries. The rationalization stems from the low and unstable incomes that can have severe implications for low productivity and economic growth (Severini et al., 2016).

While a large number of studies focus on price stability and welfare, there are not many analyses explicitly focusing on the improvement of income following from price stabilization policy (Wang and Wei, 2019). Many studies in the context of sub-Saharan Africa and food price volatility focus on price transmission in the markets, motivated by potential adverse effects of global agri-food market prices and their movements on food security for consumers (see Brümmer et al., 2016; Bellemare et al., 2013). These studies show that the consumer welfare effects of price stabilization interventions are quite extensive. Though theoretically, the effects of price fluctuations and price support on producers' welfare are well explored (Newbery and Stiglitz, 1981, among others), empirical evidence of the effect of price support and buffer stock operations on household income in a developing country context, is limited with different results. Farmers in most sub-Saharan Africa, including Ghana, continue to witness persistently unstable income and low remunerative returns from their produce (Gitau and Meyer, 2019; Minot, 2014). Empirical evidence of which policy works is therefore needed, and this study contributes to bridging this gap.

The study focuses on maize farmers and their produce in Ghana, which constitute the target for the buffer stock operations. Maize is a critical crop in Ghana as most households depend on the commodity for their daily calorie intake, and others in the maize value chain for their livelihood. Given that the buffer stock operation in Ghana is a form of support to producers to improve their income, the question we try to answer is how the buffer stock operations, as output price support intervention, impacted the household income of smallholder farmers? Additional attention is paid to the underlying factors that drive smallholder farmer's participation in the buffer stock operations. This paper contributes to the literature on price support (Aditya et al., 2017; Allen, 2016) by providing empirical evidence on how output price support implemented via buffer stock operations impacts on income of smallholder farmers in a lower-middle-income country. We use a cross-sectional household collected from a survey of smallholder maize farmers.

The rest of the paper is organized as follows: The next section provides an overview of OPS, buffer stock, and income, and details of the BSO initiative. Section three presents the methods and estimation strategy. Section four presents the empirical results, and section five concludes.

## 2 Output price support, buffer stocks, and income

Price support is a prominent tool used by countries all over the world to help farmers hedge against income losses and to smoothen out price fluctuations (Aditya et al., 2017; Allen, 2016). With price support, a market-price stabilizing scheme in the agricultural sector guarantees farmers a stable and reasonable income (Key et al., 2017). Price support raises prices of farm produce to benefit farmers by improving their net income and the purchasing power (Key et al., 2017). These interventions serve as an incentive to farmers, allowing them to invest in the adoption of acceptable agricultural practices such as the use of improved seed varieties and fertilizers to increase productions (Aditya et al., 2017; Allen, 2016). Price support also helps to stabilize commodity prices.

Commonly, three types of price support schemes are distinguished based on output market intervention (Wright and Williams, 1988). In one, the government directly pays producers' deficiency payments' which consist of the difference between a ‘target price’ and the actual market price (Russo, 2007). The USA has used this system since the 1930s (Russo, 2007). The second scheme uses marketing orders to ensure that both the producer and market prices do not fall below some specific price levels, below which supplies will be removed from the market. Until the MacSharry reforms in 1992, this approach was used as one of more measures by the Common Agriculture Policy (CAP) of the EU for the implementation of price support to farmers in the European Union (Daugbjerg, 2003). The third scheme referred to as output price support (OPS), which is the focus of this study, is a system of agricultural marketing intervention where the government purchases farm produce from farmers at a price higher than the actual market price, providing farmers with price subsidy on their produce to support their income (Allen, 2016). This type of price support has similarities with the measure under the CAP in the EU and is common in sub-Saharan Africa (Demeke et al., 2012).

Under the CAP, output price support was used to support farmers to ensure a decent standard of living for the agricultural community (Tracy, 1989). The policy aimed to support farm incomes by stabilizing the prices of some essential farm products above world market prices. CAP interventions were driven by the belief that without support, average producer prices and farm incomes would be too low, exposing farmers to the vagaries of the world market, resulting in undesired price and income fluctuations (Tracy, 1989). Guaranteed prices were set at levels, which ensured that production would be profitable for producers (Demeke et al., 2012). The policy was successful; agricultural production in the EU increased considerably and turned the EU^1^ into a significant net exporter (Oskam et al., 2011). In the 1990s, under the MacSharry reforms, the CAP policy was reviewed, and currently, the price support program no more exists. A common tool for implementing an output price support system is buffer stock operations (Hoang and Meyers, 2015).

In a buffer stock operations (BSO) system, the government makes an open offer to buy available supplies for its stockpile at a floor price. Under such a floor price scheme, the government ensures that the producer price does not fall below the floor price. Usually, the offer is at the farm gate. If not at the farm gate, actual prices for farmers can be lower because of transportation and handling costs. Stocks are later sold out again at a ceiling price to ensure that the price stays within a particular band. The existence of governmental stocks keeps market prices low, and selling at a relatively low price does even more so. As long as the stocks are sold out at the end of the season, this is attractive for consumers, including farmer-consumers. In sub-Saharan countries, buffer stock operations are commonly associated with cereals, especially maize and rice (Minot, 2014).

Smallholder farmers in developing countries, for lack of storage, are often under pressure to sell their produce right after harvest and sometimes become buyers in the latter part of the year. This situation especially applies to farmers of staple foods such as maize and rice. Smallholder farmers are compelled to undertake such action due to household demand for cash to meet short term needs and the lack of storage facilities (Hossain, 1993). With high supply after harvest, farmers receive low prices for their produce. Similarly, during lean periods, prices are high due to slow adjustment on the supply side. As a result, the agri-food system, especially in sub-Saharan Africa, is characterized by significant seasonal price variations: low prices during glut and harvest period and high price during lean (Gilbert et al., 2017). High food prices, for instance, decrease the purchasing power of the smallholder farmers and urban consumers as well. Smallholder farmers often spend a large share of their low income, about 60%, on food (Minot, 2014).

The theoretical underpinning of buffer stock operation (BSO) is that, if a government agency purchases grain or cereals when prices are low to build up stocks and sell stocks when prices are high, income would be redistributed between producers and consumers based on the sources of price variation (Antonaci et al., 2014; Fertő, 1995). The concept of buffer stock operation stems from the low prices that characterize a commodity market during the main season (glut) and the high prices during the off-season creating price fluctuations (Hossain, 1993). However, farmers are ‘impatient’ and want to sell their produce just after harvest, and the management of the supply of farm produce by the government, through purchases, stabilizes the price (Saini and Gulati, 2015). Once the government acquires the stocks, the market responds by structurally depressing prices, as traders will know that the stocks will be brought to the market in the future. In the past, this happened with skimmed milk powder stocks in the EU: the market prices remained low until the government had sold all its stocks (Jongeneel et al., 2018). The process implies that the operations have to be concluded within one season; otherwise, the prices for producers are negatively influenced.

### 2.1 The price support via buffer stock intervention in practice

In Ghana, farmers have two main options to trade their maize produce: selling to traders at the farm gate or transporting produce to the nearest market centers (Abu et al., 2016). Generally, selling at the farm gate is less remunerative due to low bargaining power and lack of price information by the farmers (Abu et al., 2016). Traders often extract rent from them by offering relatively low prices for their produce (Courtois and Subervie, 2014). However, because communities are remote with sparse road networks, selling at the market centers often involves high transaction costs. Besides, packaging costs, market tolls, and the opportunity cost of farmers' productive labour could make selling at the market centres relatively expensive (Abu et al., 2016). Even when farmers have price information, there is high uncertainty about the prices in the market centres due to the activities of ‘middlemen’ who are the interface between farmers and traders in the market centres (Fafchamps and Hill, 2005). The middlemen in the market could constitute a cartel to change the price within a short period. The aggregate effect of all these is that farmers' prices and incomes are low and uncertain.

In 2010, Ghana adopted the buffer stock operations (BSO), named National Buffer Stock Programme (NAFCO). The principal mandate of NAFCO is to guarantee a minimum price and access to the market (Benin et al., 2013). Thus, NAFCO seeks to provide market access to farmers at the farmgate with remunerative prices to enhance their incomes. The mandate also includes purchasing, selling, preserving, and distributing foodstuffs by employing a buffer stock mechanism to manage the demand and supply of the cereals. Furthermore, NAFCO is directed to manage governments' emergency food security. The operations of NAFCO place emphasis on buying from farmers rather than selling stock to consumers (Benin et al., 2013).

NAFCO uses the services of licensed buying companies (LBCs) as agents to purchase maize from smallholder farmers in rural. NAFCO pays the LBCs on a commission basis (Benin et al., 2013). The fee paid to the LBCs is determined by the addition of a margin to the fixed price at which the LBCs purchase from the farmers. The margin is based on the cost of transportation, sacks, drying, bagging, sewing, and others. The use of private business entities as LBCs is to ensure that such business entities are not crowded out in the sector due to the NAFCO (Saini and Gulati, 2015). Some of these private businesses engage in the trading of maize as they sometimes hold private stocks, though at a relatively small scale. The trading activity of private businesses is invoked since the government does not possess the logistics to carry out procurement activities in remote rural areas itself. NAFCO stores the produce and re-sells later by setting its ceiling price, devoid of interference from government, during the lean season when prices are at their peak in the open market (Wright and Williams, 1988). Although the BSO is implemented to affect prices in entire local markets, participation in BSO is optional. Farmers who opt to participate in the BSO sell their farm produce to the programme at the fixed price, whereas others opt to sell their produce to market actors in the open markets. Hence, we will also analyze the factors behind the willingness to participate in BSO.

In terms of public food stockholdings, there are three types: buffer stock, emergency stocks, and food safety net stocks for different purposes. The buffer stocks are to limit price fluctuations, maintain a floor price for producers, and to maintain a ceiling price for consumers. The emergency stocks assist vulnerable people during transitory food shortages and crises, often caused by sudden supply shocks, like natural disasters. The food safety net stock provides food for the impoverished and the chronically food insecure people (Deuss, 2015). Currently, Ghana's BSO intervention is designed to combine buffer stock and emergency stocks with a focus on maintaining reasonable floor prices for producers. Some of the stocks are sold during the lean periods to consumers, institutions, and other large-scale buyers such as poultry farmers. The remaining stocks are kept as ‘security stock’ for the emergency preparedness of the country, which is disposed at the end of the farming season and replaces with new stocks.

In theory, buffer stock operations engender three impacts on income: stabilize, reduce, or increase income (see Houck, 1973). Buffer stock stabilizes income via the management of supply-demand that offset the price movement of the commodity. This mechanism allows the floor and the ceiling prices to stay within a band to ensure stability (Abokyi et al., 2018). However, when the floor price is not reviewed over time, the market price can plummet again and cause income losses, leading to low incomes. In practice, BSO achieves increased income for farmers in two ways. First, the government mechanically fixes the minimum price based on production costs and the desired ‘optimal’ profit for farmers (Wright, 2001). In the case of the NAFCO intervention, the floor price, also called the NAFCO price, is fixed based on the crop budget (average cost of production) for the season. For NAFCO to affect farmers' income positively, the NAFCO price is set at a level substantially higher than the glut market price. In the 2011/2012 farming season, for instance, the NAFCO price was fixed such that at least a 27% profit margin could be gained by the farmer when she/he sold just after harvesting; the glut period (Benin et al., 2013). The insurance provides an implicit subsidy, output price support, to the farmers.

The open market prices during the glut periods leave farmers, at times, at a loss as the prices plummet to awful levels. Based on this observation, the NAFCO price is reviewed upwards annually. The effect of NAFCO on income, therefore, is most often positive. Though there are spatial differences in the cost of production due to differences in the local production and market conditions, including open market prices, there is no spatial variation in the setting of the NAFCO price. This is different from the old EU measures, where regional transport costs were compensated. In NAFCO, the price is set in one specific geographical market (all associated costs are noted, and from there, regional prices are calculated). The process implies that the impact of NAFCO will not be spatially neutral; favouring farmers in low-production cost locations, where most farmers might be concentrated, as NAFCO is targeted at the remote rural areas.

In addition, the activities of buffer stock operations are expected to be budget neutral, i.e., that the revenues from selling the outputs cover the payments to the farmers (plus the operational costs). However, NAFCO acts as a subsidy, benefiting poor farmers, providing emergency food reserves, and also supplying produce to some public secondary schools. These operations are financed by the government to ensure the operations of NAFCO are successful.

Second, BSO controls the market supply through purchasing and storing the excess maize to artificially increase the demand for the produce. Moreover, as prices become stable as a result of BSO, farmers are motivated to invest in increased production to increase their income (Tolley et al., 1982). The investment effect is primarily created because the system gives certainty for a more extended period. Farmers are assured of stable prices and income, encouraged to invest in the expansion of their farm, adopt fertilizer, and other modern inputs and technologies to increase outputs and income (Benin et al., 2013). Again, NAFCO enables farmers to increase the cultivation and rearing of animals as the marketing of produce no longer takes more of their time. Farmers can put the time afforded by NAFCO operations into off-farm income-generating activities. In summary, buffer stock operations, if well managed, stabilize prices as well as farmers' incomes, create market access and impact on trading, provide farmers with remunerative prices, and improved income.

### 2.2 Determinants of participation in buffer stock operations

Participation in the BSO, in general, could be potentially influenced by some underlying drivers: factors likely to influence one's choice or willingness to participate. Theoretically, these factors could lead to confoundedness and bias the estimates (Ren et al., 2018). We discuss the potential drivers we believe will influence participation in NAFCO below:

#### 2.2.1 Age of household and years of farming experience

The age of the household head could affect the household's participation in the market (see, for example, Maspaitella et al., 2018). It is observed in Kenya that smallholder farmers' participation in various markets, i.e., the sweet-potato market, is influenced by age (Mutai et al., 2013). Older people were found not to participate in this market due to a lack of information. With older farmers not having more market information, there are likely to sell their produce at the farm gate. Hence, we posit that older people are more likely to participate in the BSO initiative.

#### 2.2.2 Gender and marital status

Based on the Ghanaian culture, women are more responsible for the marketing of farm produce than men (Drafor et al., 2005). As a result, they probably have better market information on prices, the sales process, and market outlets than men. This gender role of women makes men not to be interested in the sale process but arrogate this role to their wives or any woman available to them. The women, however, prefer to sell at market centres as they anticipate higher prices, compared to men, who prefer selling at the farm gate; when men take active roles in the sales process. Therefore, women are less likely to participate in BSO than men because they have more information options on market outlets at different prices (Drafor et al., 2005). Also, the decision to sell maize to the buffer stock project could be a joint decision of both wife and husband. However, both may differ in their views, and the differing views may reduce the probability of households participating in the programme (Musah et al., 2014). Similarly, the effort of both husband and wife could provide several outlets to sell their products and hence reduce the probability of selling to the buffer stock operations.

#### 2.2.3 Education

Education is a vital determinant of awareness of programs and existing technologies. Educated farmers have a relatively high ability to obtain and process information about the projects, including NAFCO. Higher education will lead to an increased potential to acquire market information from various sources such as the media about the NAFCO program (Maspaitella et al., 2018; Lapar et al., 2003). Educated farmers, therefore, are more likely to participate in the BSO due to their informed knowledge about NAFCO.

#### 2.2.4 Access to market

In the rural areas of Ghana, where maize growing is predominant, the roads are generally not in good condition. The poor roads make access to market centres difficult, potentially influencing the level of participation in NAFCO. With increased market access, farmers have more opportunities to sell their produce, and this may present them with more options to choose from (Barrett, 2008). We, therefore, expect that farmers in the rural areas, where maize production is high, are more likely to sell their maize to NAFCO due to low market access.

#### 2.2.5 Packaging cost

Maize farmers also incur packaging costs as they sell the produce in the market centres. The cost includes packaging materials such as sacks, measuring cans, and ropes used for packaging maize. The cost may also include measuring instruments such as scales and containers. Farmers also pay market tolls for each packaged sack of maize. This packaging cost affects market participation negatively (Osebeyo and Aye, 2014). The negative effect stems from farmers not be able to afford the packaging cost and often sell at the farm gate (Fafchamps and Hill, 2005), increasing their likelihood to participate in the BSO initiative.

#### 2.2.6 Transport cost

Transport cost is a significant determinant in market participation (Osebeyo and Aye, 2014). High transport costs deter the entry of smallholder farmers into the market for agricultural commodities (Osebeyo and Aye, 2014). Therefore, as transportation cost increases, farmers will likely look for alternative means to sell their products, and hence increase the likelihood of their participation in the BSO initiative.

#### 2.2.7 Access to extension services

Access to extension services provides farmers with additional market information about the NAFCO initiative through farmer associations. Belonging to a farmer association, therefore, increases the farmer's access to extension services. The improved access to market information about NAFCO could ultimately influence awareness about the program (Barrett, 2008). The expectation, therefore, is that access to extension services increases the probability of smallholder farmers' participating in the BSO initiative.

**Figure 1:**
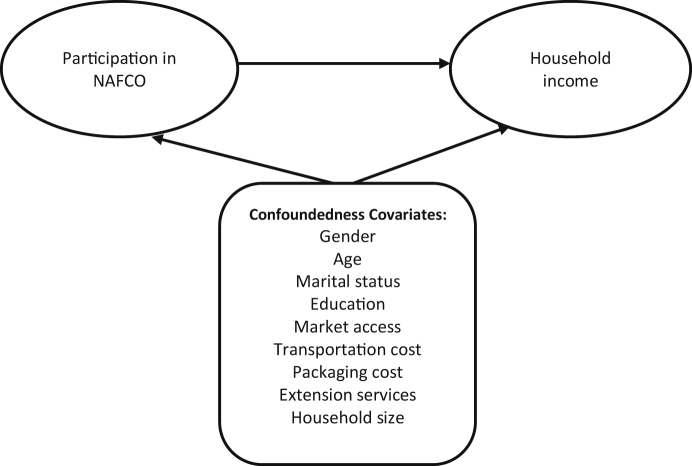
The confoundedness between participation in BSO and household income.

We summarise the confoundedness between participation in the BSO and its covariates discussed above in Figure 1 below:

### 2.3 Outcome variables

In this study, one of our outcome variables is household income. Rural farm households depend on different sources of income, including farm and non-farm activities. In rural Ghana, farm income constitutes a significant portion of smallholder household's income (Baidoo et al., 2016). Though smallholder farmers' income in Ghana is generally composed of crop income, livestock productions, and non-farm sources, our income indicator (variable) is limited to the component of household income that is directly affected by the BSO. We assume BSO may not affect the non-farm income directly: because farmers' agricultural (farm) activities are diversified, and any labour saved from selling to NAFCO is more likely to be used on other farm activities such as livestock, vegetable production. In rural areas of low-income countries, household farm income, relating to cash received alone, is unduly restrictive because farmers keep part of their produce for the household's consumption, as gifts to other family members, or even to pay farmworkers' wages in kind. Therefore, for an adequate concept of income, and calculation sake, farm income of a household is the value of crops sold, plus crops used for own consumption, in-kind payments, gifts valued at market prices minus the costs for all inputs and hired labour (Wordofa and Sassi, 2017).

Another outcome variable that is estimated is *farm income per output:* farm income divided by total output. The aim of estimating this variable is to reduce variations in farm income caused by variations in outputs. The outcome variables, determining the participating in BSO, are *total farm income* and *farm income per output.* Thus, we test the central hypothesis as output price support via buffer stock operations has a positive effect on total farm income and farm income per output.

## 3 Methodology

### 3.1 Data

The survey on which we draw our data is from smallholder farmers' households in the policy-on and policy-off areas in five maize growing districts of Ghana. The policy-on areas were determined at the onset of the NAFCO initiative. Based on the availability of warehouses for storing produce, maize growing areas with enough warehouses were earmarked for the initiative as policy-on and those without warehouses as policy-off areas.

The goal of the survey was to collect household data from the participant and non-participant farmers in the BSO initiative to elicit information on household income, marketing of farm produce, and others. A stratified random sample of 507 smallholder farmers was selected at three distinct spatial scales: (1) districts, (2) communities/villages, and (3) household addresses. At the first stage, five districts were selected out of 20 major maize producing districts: Nkoranza South, Nkoranza North, Ejura Sekyere Kajebi, and Jasikan; 3 districts were selected as the policy-on areas and two districts as the policy-off areas. Our choice of the five districts is based on their characteristic being among the most dominant maize producing districts in the policy-on and policy-off areas and constitute among the maize breadbasket of the country. In addition, the five districts are among the top largest maize production in terms of volumes are quite similar in their maize production volumes, with smallholder farming being predominant. The five districts also cover a wider geographical spread of maize production in the country. Hence provide a good representation of smallholder farmers in Ghana. A total of 90 rural maize growing communities were identified at the first stage. At the second stage, we randomly selected 40 rural maize communities (villages) out of the 90 communities. At the third stage, approximately 13 households were randomly selected out of the sample of 40 villages. Information on household demographics, production levels, produce marketing, and income levels were elicited from the household head. Households with incomplete questionnaires due to non-responses were dropped, leaving a total of 507 out of 520 households. Before the survey, a total of 252 participants and 268 farmers were targeted.

Table 1 below presents the description of the variables and their measurements.

**Table 1:** Summary of variables.

No	Variable	Variable Description	Measurement
1	*NAFCO*	Buffer stock farmer	1 = NAFCO, 0 = non NAFCO
2	*Ln(TFInc)*	Total Farm income	Natural log of total income from farm activities in Ghana Cedis
3	*Ln(FInc/U)*	Farm income/unit output	Natural log of farm income per unit output in Ghana Cedis
4	*HS*	Household size	Members in the household
5	*Ln(Age)*	Age	Natural log of the age of household head in years
6	*Gen*	Gender	Gender of the household head
7	*Mar*	Marital status	Marital status of the household head
8	*Edu*	Education	Education of head of household
9	*Market*	Market Access	1 = access to market, 0 = no access
10	*Ln(Expr)*	Farming experience	Natural log of years of working as a farmer
11	*Ext*	Extension service	1 = access to extension, 0 = no access
12	*Ln(Trans)*	Transport cost	Natural log of cost of transporting produce to market
13	*LnPack*	Packaging cost	Natural log of cost of packing produce for sale in

Note: Ln is a natural log. The income and cost variables are in Ghana Cedis (GH¢). GH¢1 is equivalent to USD 0.21 as of August 2018.

A summary of the descriptive statistics of the variable in Table 1 is presented in Table 2. Substantial differences can be observed in the income variables between the treated and the control farmers: 2,200.26 and 441.22 versus 1,028.73 and 218.29. In Table 2, the *mean* costs of transportation to market places and packaging of produce for the control groups are 49.19 and 51.09, respectively. These are marginally lower for those in the treated group: 57.15 and 52.77. To ensure that the distribution of the data is not skewed, we applied the log transformations to the data to approximately conform the data to normality. The log transformation reduces the influence of the outliers and normalizes the distribution of the income variables to increase the validity of the associated statistical analyses.

**Table 2:** Variable description and summary of descriptive statistics.

No	Variable	Policy-off (Control, *N* = 257)				Policy-on (Treated, *N* = 250)				Pooled (*N* = 507)	
		Mean	SD.	Min.	Max.	Mean	SD.	Min.	Max.	Mean	SD.
1	*NAFCO*	1	1	1	1	0	0	0	0	-	-
2	*TCInc*	1,028.73	1,152.04	100.00	10,000.00	2,200.26	2,624.01	200.00	15,000.00	16,06.41	2,098.45
3	*CInc/U*	218.29	337.52	8.33	3000.00	441.22	467.33	25.00	3000.00	328.21	421.37
4	*Ln(TFInc)∗*	6.52	0.89	4.61	9.21	7.27	0.86	5.30	9.62	6.89	0.95
5	*Ln(FInc/U)*	4.83	1.01	2.12	8.01	5.70	0.87	3.22	8.01	5.26	1.04
6	*HS*	5.90	2.32	1.00	13.00	5.58	2.27	1.00	13.00	5.75	2.30
7	*Ln(Age)*	3.85	0.28	3.00	4.48	3.88	0.19	3.22	4.32	3.86	0.24
8	*Gen*	0.67	0.47	0.00	1.00	0.78	0.41	0.00	1.00	0.73	0.45
9	*Mar*	0.80	0.40	0.00	1.00	0.76	0.43	0.00	1.00	0.78	0.41
10	*Edu*	2.29	0.92	1.00	5.00	2.42	0.88	1.00	5.00	2.36	0.90
11	*Market*	3.05	0.74	1.00	5.00	2.84	0.71	2.00	4.00	2.94	0.73
12	*ln(Expr)*	3.23	0.83	0.00	4.48	3.33	0.54	1.10	4.32	3.28	0.70
13	*Ext*	3.82	0.55	2.50	6.08	4.27	0.28	3.58	5.42	4.04	0.49
14	*ln(Trans)*	3.81	0.45	2.75	4.96	4.01	0.29	2.79	4.94	3.91	0.39
15	*Ln(Pack)*	3.86	0.47	0.69	5.00	3.95	0.19	2.30	4.61	3.91	0.36

Note: The policy-on are the BSO farmers, and the policy-off are the non-BSO farmers. ∗ Ln indicates the log transformation of the TFInc and FInc/U. The application of the log transformation of the data reduced the influence of the outliers and normalized the distribution of the income variables to increase the validity of the results.

Generally, the average educational level, age, marital status, and household size are similar among the treatment group and the control group (non-participants). Access to extension services, transportation costs, and packaging costs are higher for the treated group than the control, with market access, the reverse holding (see Table 2).

### 3.2 Methods

The main challenge in assessing the impact of non-experimental data is how to overcome selection bias and attribute changes in income associated with the target group (smallholder maize farmers) to the NAFCO initiative (Datta, 2015). The impact could be measured by the difference between the expected value of income earned by household *j* in the target group and the expected value of income that the households would have received if it had not participated in the initiative (Benin et al., 2013). This difference is the impact of the initiative; the average treatment effect of the treated ($ATT_{j}^{i}$):

(1) $$ATT_{j}^{i}=E\left[LnInc_{1j}^{i}|NAFCO_{j}^{i}=1]-E[LnInc_{o\;j}^{i}|NAFCO_{j}^{i}=0\right]$$

where BSO is a dummy variable; with *1* indicating participation in the buffer stock operation and *0* indicating otherwise, *LnInc* represents household income variable; *Ln(TFInc) or Ln(FInc)*, *E[.]* is the expectation operator, $LnInc_{1j}^{i}|NAFCO_{j}^{i}=1$ representing the average household income for the treated group and $LnInc_{1j}^{i}|NAFCO_{j}^{i}=0$ representing the average household income for the counterfactual (the control group).

#### 3.2.1 Coarsened exact matching (CEM)

We adopted the Coarsened Exact Matching (CEM) and the Propensity Score Matching (PSM) in our empirical approach to construct a control group from the non-participant that is closely similar to the treatment group of household farmers (Iacus et al., 2012). CEM mitigates selection bias and heterogeneity by reducing the level of imbalance. CEM further controls for selection bias by eliminating non-analogous observations in the treatment and control groups (David et al., 2013). CEM, therefore, offers a solution to this problem by creating “statistical twins,” one with and one without the treatment, and ensure comparability of the groups. CEM also mitigates possible endogeneity bias because the matching reduces the differences in the observable characteristics between observations in the treated control groups (Luo and Escalante, 2017).

Our motivation for using the CEM is that the CEM allows a balance between the policy-on and the policy-off group to be chosen ex-ante rather than being revealed through an iterative process of ex-post balance checking (Nilsson et al., 2019). The balance between the treated and the control groups is chosen ex-ante, reducing model dependence. The choice also ensures that error in the estimation of the treatment effect is minimized. In the CEM algorithm, treated households and control households are exactly matched, after the coarsening of the variables (see Iacus et al., 2011). Coarsening is the recoding of a variable so that substantively similar values are grouped and assigned the same numerical value according to the researcher's ideas, with the matching procedure generating weights^2^ that are used in the subsequent regressions to estimate the treatment effect (see Iacus et al., 2012).

Coarsening leads to a set of strata of the original outcomes. Within each stratum, the treated farmers are matched with those in the control groups. Unmatched farmers in the control group are discarded (Nilsson et al., 2019). The success of the matching is measured by the multivariate imbalance measures *L*_*1*_; that is, the distances in covariate values between the treated and control before and after the matching are compared with a reduction indicating success. In specific, the *L*_*1*_ statistic is a measure of the overall imbalance based on the multivariate distribution of all the pre-treatment covariates and their interactions defined as (Iacus et al., 2011):

(2) $$L_{1}\left(f,g\right)=\sum_{\ell_{1.....}\ell_{k}\in H\left(X\right)}\left|f_{\ell_{1}.....\ell_{k}}-g_{\ell_{1}.....\ell_{k}}\right|$$

where $f_{\ell_{i}.......\ell_{k}}$ and $g_{\ell_{i}........\ell_{k}}$ denote the relative frequencies for the treated and untreated units. *L*_*1*_ ranges from 0 to 1, with higher values denoting more imbalance. *H(X)* is the set of values generated by coarsening from the set of continuous variables *X*, with binary and categorical variables retaining their original values (Dooley et al., 2014). Note that for the pre-matching, the H(X) is just the original values of X. The measure in Eq. (2) can also be quantified for each variable *j* separately, i.e., univariate imbalance measure (*I*_*1*_), for assessment of variable-specific imbalance (Corneo et al., 2010) as:

(3) $$I_{1}^{j}={\bar{X}}_{m_{T},w}^{j}-{\bar{X}}_{m_{C},w}^{j}\;j=1,2,....k$$

where *m*_*T*_*, m*_*C*_ are the average mean for matched treated and matched control units, respectively, w is the weight assigned to each unit during the CEM matching.

From Eq. (3), the I_i_ is the difference in the means of variable *j* for the group of treated (*m*_*T*_) and control units (*m*_*C*_) matched, weighted by the matching weights assigned to each unit. We use gender, marital status, education, and household size as the matching variables as these variables influence participation in the BSO (Musah et al., 2014).

#### 3.2.2 Estimation of treatment effect

We estimate the treatment effect with a weighted regression because the CEM application generates weights. Iacus et al. (2012) demonstrate that the inclusion of control variables in the regression, including those used in the matching, can control for the remaining heterogeneity between the groups since it is impossible to account for all the heterogeneity fully. So, we estimated Eq. (1) by weighted least square (WLS) regression as:

(4) $$LnInc_{i}^{\prime }=\beta_{0}^{\prime }+\beta_{1}NAFCO_{i}^{\prime }+\beta_{2}LnAge_{i}^{\prime }+\beta_{3}Mar_{i}^{\prime }+\beta_{4}Gen_{i}^{\prime }+\beta_{5}Edu_{i}^{\prime }+\beta_{6}HS_{i}^{\prime }+\beta_{7}LnExp_{i}^{\prime }+\varepsilon_{i}$$

where the β_0-7_ denotes the coefficients to be estimated with β_1,_ indicating the impact of the initiative. The variables are as defined in Table 1 earlier; *Inc* denotes the dependent variable of household *i* (total farm income or farm income/output); control variables include the variables used in the coarsening; *ε*_*i*_ is the error term. The asterisk on the variables in Eq. (4) indicates that the variables are weighted using the assigned CEM weights.

#### 3.2.3 CEM combined with propensity score matching (PSM) analysis

Even though CEM is preferred to propensity score matching (PSM) due to the latter's tendency to increase the imbalance between the two different groups, control and treated (Nilsson et al., 2019), a combination of the two approaches, i.e., CEM preceding PSM was used (King and Nielsen, 2019), our primary analysis, therefore, involves two lines of modelling; CEM and CEM combined with PSM. The CEM, combined with PSM, means that the PSM analysis is restricted to the CEM matched data (Blackwell et al., 2009). Instead of using control variables to control the remaining heterogeneity, we follow Iacus et al. (2012), a study that observed that combining PSM analysis with CEM can further reduce the imbalance in the data and may perform better in terms of reducing imbalances than the CEM alone (see, for example, Wells et al., 2013).

The PSM technique estimates the propensity score (probability) to be treated for each treated and untreated individual and matches treated individuals with one or multiple of the untreated individuals who have the same or a similar propensity score (Ren et al., 2018). The PSM technique works on two assumptions (Owusu et al., 2011): common support and conditional independence assumptions. The common support assumption posits that individuals in the treated and control groups must have a positive probability, within 0 and 1, of belonging to either group, and the distribution of the probabilities across the groups must be such that comparable individuals can be found across the groups. Also, the conditional independence assumption states that for a given set of covariates, participation in the treatment is independent of potential outcomes.

In terms of techniques, the PSM involves three main steps; estimating the propensity scores, matching to estimate the average treatment effect on the treated *(ATT),* and assessing the quality of the matching (Abbay and Rutten, 2016). In our estimate, we first defined the probit model for estimating the propensity scores for a farmer *i* as:

(5) $$\begin{array}{l} NAFCO=\alpha_{0}+\alpha_{1}Gen_{i}+\alpha_{2}LnAge_{i}+\alpha_{3}Mar_{i}+\alpha_{4}Edu_{i}+\alpha_{5}Market_{i}+\alpha_{6}LnPack_{i}+\\ \alpha_{7}LnTrans+\alpha_{8}Ext_{i}+\varepsilon_{i} \end{array}$$

where *NAFCO* is a binary variable with 1 as participation in BSO and 0 being otherwise, the other variables are as defined in Table 2.

In the second step, the subject in the treatment group is matched with those in the control group based on the similarity of their propensity scores to estimate the impact. The PSM estimator of the *ATT* is the difference in the outcomes between the treatment and the control groups, matched appropriately by their propensity scores (Abbay and Rutten, 2016). There are several methods to do the PSM matching, including the kernel, nearest neighbor, caliper, and radius matching. We used the Kernel, Nearest Neighbour, Caliper, and the Radius matchings to estimate the *ATT*.

Econometrically, a balance test assesses the quality of the matching of PSM analysis using a two-sample t-test (Becerril and Abdulai, 2010). If a balance is achieved, there would be no significant difference between the covariate means of the treatment and the comparison groups after the matching. We use three metrics to assess the quality of the matches: the mean absolute standardized bias (MASB), pseudo *R*2^*,*^ and the p-values of the likelihood ratio test, a test of the joint significance of all the covariates before and after the matching (Becerril and Abdulai, 2010). The MASB is a standard metric used to assess the quality of the matching (Rosenbaum and Rubin, 1985). MASB is the weighted difference in means divided by the standard deviation in the original full comparison group (Rosenbaum and Rubin, 1985). MASB is the overall measure of covariate imbalance, and a MASB of less than 20% is an indication success of the matching.

## 4 Empirical results and discussion

The results of our empirical estimation are presented in two parts. First, we present the matching results and, second, the results of the PSM analysis. The results of the PSM analysis discuss the impact of the policy on farmers' income as well as a sensitivity analysis for possible hidden biases.

### 4.1 The matching results

Table 3 presents the results of the CEM matching, and the results are in two parts: the imbalances based on the raw data and results after coarsening the variables.

**Table 3:** The matching results.

A: Raw data								
Variable	Mean control group	Mean treated group	The difference in means (I_1_∗)	The between-group mean differences in means by quantile^3^				
				Min	25%	50%	75%	Max
Gen	0.67	0.78	0.108	0	1	0	0	0
Mar	0.80	0.76	-0.033	0	0	0	0	0
Edu	2.29	2.42	0.131	0	0	1	0	0
HH	5.90	5.58	-0.326	0	0	-1	0	0
Multivariate imbalance measure L_1∗∗_	0.442	-	-	-	-	-		

B: After coarsening								
Variable			The difference in means (I_1_∗)	The between-group mean differences in means by quantile				
				Min	25%	50%	75%	Max
Gen			1.20e-16	0	0	0	0	0
Mar			1.80e-16	0	0	0	0	0
Edu			1.40e-17	0	0	0	0	0
HH			0.05453	1	0	0	0	0
Multivariate imbalance measure L_1∗∗_	0.114	**-**	-	**-**	**-**	**-**		

			Policy-off (0)	Policy-on (1)	Pooled			
All obs.			257	250	507			
Matched			184	202	386			
Unmatched			73	48	121			
The number of strata^4^:					100			
Number of matched strata					44			

∗∗Multivariate imbalance measure estimated from Eq. (2), ∗Univariate imbalance measure estimated from Eq. (3).

By comparing the pre and post-match imbalance measures, the *L*_*1*_ *statistics* for the raw and the matched data in Table 3, the results indicate that imbalance is reduced from 0.442 to 0.114 (by a factor of 3.9). The *I*_*1*_ measures for the specific variables also show a similar trend of reduction in imbalance. For instance, the *I*_*1*_ for gender reduced from 0.108 to1.20e-16. Overall, the results mean that the matching reduced the heterogeneity between the treated and the control households not only in the means but also in joint distributions of the data (Nilsson et al., 2019). While there is no generally acceptable level of benchmark for *L*_i_, Firestone (2015) recommended 0.2 as acceptable levels. Therefore, with an *L*_*1*_ measure of 0.114, we conclude that beneficiary households are comparable to the non-beneficiary group and are a valid and appropriate counterfactual group for estimation of the treatment effect.

### 4.2 Results of the probit model of the PSM analysis

After carrying out the Coarsened Exact Matching, a propensity score matching analysis was performed on the CEM data via a probit model to identify the determinants that influence the probability of farmers participating in the buffer stock initiative based on Eq. (5). The results are presented in Table 4. The p-value for the Chi^2^ for the overall model presented in Table 4 is statistically significant at 1%, and the pseudo-*R*^*2*^ both indicate quite a good fit.

**Table 4:** The propensity score probit model.

Model (1)	
Variables	Coef.
*Gen*	0.180 (0.238)
*Ln(Age)*	1.250∗∗∗ (0.350)
*Mar*	-0.396 (0.276)
*Edu*	-0.016 (0.096)
*Market*	-0.407∗∗∗ (0.110)
*Ln(Pack)*	0.871∗∗∗ (0.270)
*Ln(Trans)*	0.765∗∗∗ (0.226)
*Ext*	1.785∗∗∗ (0.195)
*Ln(Exp)*	0.154 (0.122)
*HS*	-0.119∗∗∗ (0.040)
*Cons*	-16.878∗∗∗ (2.216)

LR chi^2^	165.79∗∗∗
Pseudo R^2^	0.316
Log-likelihood	-179.22
Obs.	379

∗∗∗ are values statistically significant at 1%. The standard errors are in parentheses.

The results of the probit model reveal that participation in the programme is influenced by *age, access to the market, packaging cost, transport cost, and access to extension services*. The results show that *age* is positively associated with participation in the BSO initiative. Further, results show that *market access* has a negative association with NAFCO, indicating increased access to market outlets makes farmers less likely to sell their produce to NAFCO. The estimated coefficients of *transport and packaging costs* are positive and significant at 1%, indicating a positive association with these variables to participation in BSO. The results mean that as transport and packaging costs increase, farmers are more likely to sell to NAFCO instead of transporting their produce to distant markets. The variable *extension* also has a similarly positive and significant relationship with the BSO initiative, at a 1% significant level. The result shows the importance of extension services to participating in the BSO initiative. *HS* (household size) has a significant negative relation with participation in the BSO initiative, indicating that large households are more likely not to sell their produce to the initiative. Large households are likely to have more labour to carry out their produce to various market outlets for sales compared to smaller households.

### 4.3 Impact of buffer stock operations on income

In assessing the impact of BSO on income, we first apply the Durbin–Wu–Hausman (DWH) test for endogeneity on the data. Even though we initially expected endogeneity between income and NAFCO, the Durbin-Wu-Hausman test statistics, restricted to the CEM data, do not confirm a reverse relation between the two variables to warrant the use of instrumental variable estimation (see details in Table A2). The coefficients of 0.415 and 0.941 for the predicted residual for both *Ln(TFInc) and Ln(TFinc/U)* are insignificant, indicating that there is no statistically significant endogeneity of NAFCO for both the *Ln(TFInc) and TFinc/U* dependent variables. These results imply that CEM effectively mitigates potential endogeneity (Luo and Escalante, 2017).

The impact of buffer stock operations on household income (treatment effect) is estimated via two approaches: Weighted least square (WLS) regression and CEM-based PSM estimation. The results for the WLS estimates are presented in Table 5 and are for the two outcome variables: total farm income (*TFInc*) and farm income per unit output *(FInc/U).* We also present the results for the unmatched and matched data.

**Table 5:** Estimated impact on household income, WLS estimates.

Model (2)				
Variables	Unmatched data		Matched data	
	Total income	Income/output	Total income	Income/output
*Treatment(NAFCO)*	0.700∗∗∗(0.076)	0.776∗∗∗ (0.078)	0.805∗∗∗ (0.084)	0.834∗∗∗ (0.086)
Ln (Age)	-0.158 (0.166)	-0.404∗∗ (0.169)	-0.422∗∗ (0.188)	-0.579∗∗∗ (0.192)
*Mar*	0.043 (0.098)	-0.035 (0.100)	-0.070 (0.134)	-0.067 (0.138)
*Gen*	0.217∗∗ (0.091)	0.167∗ (0.093)	0.407∗∗∗ (0.128)	0.340∗∗∗ (0.132)
*Edu*	0.232∗∗∗ (0.042)	0.233∗∗∗ (0.043)	0.214∗∗∗ (0.054)	0.217∗∗∗ (0.055)
*HS*	0.0137 (0.017)	-0.137∗∗∗ (0.017)	0.059∗∗∗ (0.022)	-0.114∗∗∗ (0.023)
*Ln(Expr)*	0.090 (0.056)	0.089 (0.057)	0.076 (0.062)	0.088 (0.064)
*Cons*	6∗∗∗ (0.627)	6.291∗∗∗ (0.637)	6.781 (0.691) ∗∗∗	6.713∗∗∗ (0.714)

R-square	0.226	0.321	0.253	0.306
F-stat	20.84∗∗∗	35.15∗∗∗	18.30∗∗∗	23.76∗∗∗
RMSE	0.840	0.854	0.814	0.832
Obs.	506	506	386	386

Notes: the dependent variables are the natural log of total farm income, Ln(TFInc), and the log of farm income/output, Ln(TFinc/U); ∗∗∗, ∗∗, ∗; are values statistically significant at 1%, 5%, and 10%, respectively. The standard errors are in parentheses.

As shown in Table 5, the results of the estimated coefficients of the treatment variable, *NAFCO*, which measures the impact (ATT) for both models, are significant at a 1% level, indicating that participation in the BSO affects income. The coefficient shows how much more income a farmer gets, on average, if he/she participates in the BSO. To calculate the amount in percentage terms, the treated farmers get compared to the control farmers, the estimated coefficient (ATT) is compared to the descriptive statistics of the control group, i.e., ATT divide by the mean income of the control group. The results show that the treatment effects *(ATT)* are 0.805 and 0.834 for *Ln(TFInc)* and *Ln(TFinc/U),* respectively. By comparing the results of the *ATT* with the mean values of 6.52 and 4.83 for the control group (see Table 2 for descriptive statistics), the results indicate that participation in the buffer stock operations improves the total farm income and the farm income/output of the treated farmers by 12.33% and 17.27%, respectively.

The control variables, *age, gender, education, household size*, are significant at 1%, providing further assurance that these variables are appropriate as control variables. The results for the control variables in Table 5 further show that *age* is associated with a negative impact on both *Ln(TFInc)* and *Ln(TFinc/U)* variables. The negative effect of age is due to its negative relationship with participation in the BSO, as established earlier in section 4.

Furthermore, positive relationships are found for *gender* and *income*, implying that men, who participate in the BSO, increase their income more as compared to women. A positive relationship between BSO and income is also reported for *education.* The estimates for *household size* show that this control variable has a significant impact on both income variables. However, while the effect is positive for the *total farm income*, that of *farm income/output* is negative. The reason for this may be that the variable *farm income/output* has been normalized by output. *Household size* is a crucial determinant of farm output, therefore normalizing the income variable reduces bias due to *household size*.

**Figure A1:**
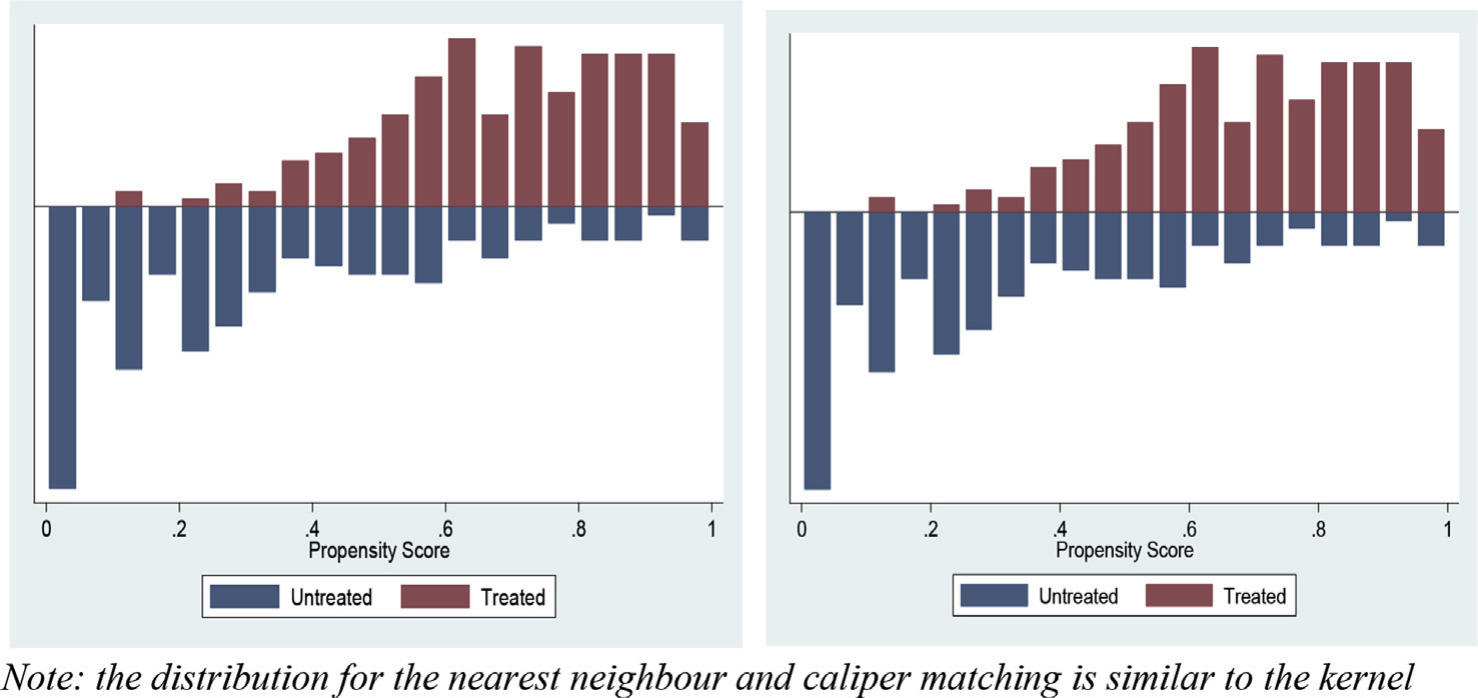
Propensity score density distribution for (a) kernel and (b) radius matchings.

Tables 6 and 7 discuss the CEM-PSM results. The indicators of matching-quality for the CEM-PSM analysis presented in Table 7 show a substantial reduction in mean absolute bias for all covariates, which underlines that the PSM matching is successful (for further details, see Table A1). The mean absolute standard bias (MASB) provides a global comparison of the balance across matching algorithms. Further, the results of the different matching algorithms yield similar results, qualitatively, and quantitatively. The post-match MASB results ranging from 6.30%-7.60%, are far lower than the 20% benchmark recommended by Rosenbaum and Rubin (1985). Besides, pseudo-R^2^ values after matching are relatively low, indicating the success of the matchings. A histogram showing the distribution of the estimated propensity scores is presented in Figure A1. Visual examination of the histograms reveals that there is a substantial overlap of the distribution of the scores for both the treated and the control groups indicating that the common support condition is satisfied, implying that the overall matching procedure was successful.

**Table 6:** The impact of CEM-PSM analysis.

Model (3)							
Matching	Variable		BSO (Treated)	BSO (Control)	Difference (ATT)	% Difference	Obs.
KN	Total farm income	Unmatched	7.329	6.532	0.797∗∗∗ (0.088)	-	379
		ATT	7.329	6.251	1.078∗∗∗ (0.133)	17.25%	
	Farm income/output	Unmatched	5.695	4.814	0.882∗∗∗ (0.093)	-	
		ATT	5.695	4.578	1.118∗∗∗ (0.145)	24.42%	
NNM	Total farm income	Unmatched	7.329	6.532	0.797∗∗∗ (0.088)	-	
		ATT	7.329	6.348	0.982∗∗∗ (0.163)	15.50%	
	Farm income/output	Unmatched	5.695	4.814	0.882∗∗∗ (0.094)	-	379
		ATT	5.695	4.720	0.976∗∗∗ (0.168)	21.00%	
CM	Total farm income	Unmatched	7.329	6.532	0.797∗∗∗(0.088)	-	
		ATT	7.329	6.348	0.982∗∗∗(0.162)	15.50%	379
	Farm income/output	Unmatched	5.695	4.814	0.882∗∗∗(0.093)	-	
		ATT	5.695	4.720	0.976∗∗∗ (0.163)	21.00%	
	Total farm income	Unmatched	7.329	6.532	0.797∗∗∗(0.088)	-	
		ATT	7.329	6.272	1.057∗∗∗(0.146)	17.00%	379
RM	Farm income/output	Unmatched	5.695	4.814	0.882∗∗∗(0.094)	-	
		ATT	5.695	4.596	1.100∗∗∗(0.157)	24.00%	

Notes: The dependent (outcome) variables are the natural logs of total farm income, and the log of farm income/output ∗∗∗ represents significant at 1%; KNM-kernel matching, NNM- nearest neighbour matching; CM-caliper matching, RM-radius matching. The standard errors are in parentheses.

**Table 7:** The balance test results for the CEM-PSM analysis.

	KNM		NBM		CM		RM	
	Unmatched	Matched	Unmatched	Matched	Unmatched	Matched	Unmatched	Matched
Pseudo *R*^*2*^	0.29	0.01	0.29	0.05	0.29	0.05	0.29	0.02
LR *Chi*^*2*^	196.83	7.91	196.83	28.17	196.83	28.17	196.83	10.59
P > *Chi*^*2*^	0.00	0.64	0.00	0.02	0.00	0.02	0.00	0.39
MASB %	29.70	6.30	29.70	7.60	29.70	7.60	29.70	6.90

Notes: Detailed test results are presented in Table A2 in the Appendix.

The corresponding *t*-values for both variables are significant at 1% for all the estimates by the various PSM matching algorithms, indicating the BSO has improved the income of households. The results in Table 6 also show that the Nearest Neighbour and the Caliper Matchings (see, for example, Rosenbaum and Rubin, 1985; Dehejia and Wahba, 2002) yielded similar treatment effects; 0.982 and 0.976 for *Ln(TFInc) and Ln(FInc/U)*, respectively, indicating that participating in NAFCO increases the total farm income and farm income/output of farmers by 15.50% and 21.00%, respectively. The ATT estimates from the Radius Matching also yielded estimates of 1.057 for *Ln(TFInc)* and 1.100 for *Ln(FInc/U).* The results indicate that participation in BSO improves the income variables by 16.85% and 24.00%, respectively. Lastly, the Kernel matchings produce ATT estimates of 1.078 and 1.118, for the *Ln(TFInc) and Ln(FInc/U),* respectively, indicating that the total farm income and the farm income/output of the BSO farmers (treated) are 17.25% and 24.42% more than the non-BSO farmers. Our results corroborate the findings by Wang and Wei (2019) that noted that output price support policy instruments, implemented through buffer stock operations, improve farm income.

The results from the WLS and CEM-PSM are broadly similar, though the CEM-PSM estimates are marginally higher than the WLS estimates. For instance, the WLS estimates show ATT of 0.805 (12.33% improvement) for *Ln(TFInc)* compared to the CEM-PSM (NNM) estimate of 0.982 (15.50% improvement) for the same variable. In the case of *Ln(FInc/U),* the WLS estimates yielded 0.843 (17.27% improvement) versus 0.976 (21% improvement) for the CEM-PSM (NNM) estimate. The results show that output price support (OPS) implemented via the buffer stock operations (BSO) intervention has positively impacted on the total farm income and farm income/output of the smallholder farmers. The convergence of the WLS and CEM-PSM estimates show the robustness of our results and attests that the estimated treatment effects are valid, and the buffer stock operations (BSO) has an impact on income.

A sensitivity analysis performed on the CEM-PSM estimates against possible hidden bias produced a result (see Table 8) showing that the *ATT* is statistically significant even at high levels of gamma (3.0), implying that the estimated *ATT* is substantially free of hidden bias (Duvendack and Palmer-Jones, 2012). A sensitivity analysis checks the estimates against hidden bias, i.e., assess whether the inferences about effects of participation in the BSO may be altered by unobserved variables (unmeasured confounder) (Warsanga and Evans, 2018; Becker and Caliendo, 2007).

**Table 8:** Results of the sensitivity analysis for radius PSM matching estimates.

Gamma (Γ)∗	Total farm income				Farm income/unit			
	Significance level (Wilcoxon signed-rank test)		Hodges-Lehmann point estimate		Significance level (Wilcoxon signed-rank test)		Hodges-Lehmann point estimates	
	Upper	Lower	Upper	Lower	Upper	Lower	Upper	Lower
1	0	0	1.007	1.007	0	0	1.102	1.102
1.5	3.50E-12	0	0.791	1.204	0	0	0.939	1.269
2	3.50E-08	0	0.641	1.354	5.40E-15	0	0.824	1.392
2.5	7.30E-06	0	0.524	1.459	6.30E-12	0	0.736	1.482
3	0.000222	0	0.438	1.557	7.10E-10	0	0.662	1.555

Note: ∗ = gamma -log odds of differential assignment due to unobserved factors.

## 5 Conclusions and policy implications

This study has assessed how buffer stock operations (BSO) affect the income of smallholder farmers' in Ghana. We used household and farm-level data with matching methods (CEM and PSM) to better compare farmers who participate in BSO and those who do not as we estimate the effect of BSO on smallholder farmers' income. The income of smallholder farmers recorded a positive effect as a result of farmers participating in the BSO initiative by the government. The results suggest that the promotion of output price support through buffer stock operations in developing economies, such as Ghana, can improve smallholder farmers' income. Farmers who participated in the buffer stock operations saw their household income improve by at least 12% and income per unit of output by at least 17%. The results further indicated that age, gender, access to market, and use of extension services, as well as transport and packaging costs, drive the participation of smallholder farmers in the buffer stock operations in Ghana. The results demonstrate that buffer stock operations improve smallholder farmers' income in a developing world setting and contribute to the agricultural policy of income stabilization: public buffer stock operation is not only a price stabilization tool but also a poverty reduction tool/instrument in low-income countries.

Even though our results show that the current BSO is quite successful, past experiences from developed countries show that public stocks can be costly and even affect other countries. For instance, under the Common Agricultural Policy, farmers in the EU received a fixed minimum price for their farm products, which helped support their incomes in the 1960s–1980s. Over time, international prices were depressed for those importing and exporting countries that could not compete for the low price of produce from the EU market, hurting those countries (Deuss, 2015). Due to storage cost and export subsidies, the policy incurred massive costs and was withdrawn by the late 1990s (Deuss, 2015). This observation has implications for Ghana, in that in the event of overproduction, NAFCO has to purchase more produce for storage to make an impact on incomes since a high-budget constraint can affect NAFCO's success negatively. In such a situation, the ceiling prices would have to be low and may not be effective.

With the above in mind, we draw two policy implications from our results. First, the results imply that well-planned public buffer stockholding is still a viable option for governments in developing countries to stabilize and improve incomes of smallholder farmers, despite its unsuccessfulness in some African countries, such as Malawi, Zambia, and Zimbabwe. In these countries, the initiative was initially successful but later had challenges due to high fiscal cost and multiple objectives (see, for example, Deuss, 2015; Brümmer et al., 2016). The success of the buffer stock operations (NAFCO) in Ghana implies that public buffer stock operations can help both producers and consumers if the emphasis is placed on one group (say targeting producers only), and credible and achievable objectives are set.

Second, the results may serve as an impetus for the upscaling of the NAFCO initiative to cover other crops and in other parts of the country. However, in any upscaling of such intervention, attention should be paid to large scale and smallholder farmers who engage in private storage since public buffer stock operations (BSO) schemes can disincentive the private sector for stockholding as literature in sub-Saharan countries shows (World Bank, 2012). When the private sector is crowded out, the government is more likely to face a more significant challenge in stabilizing prices and improving income due to more pressure on the government's budget (Gilbert, 2011). However, with transparent information on public grain stock levels, crowding out could be reduced (Gilbert, 2011). Therefore, future research could investigate how transparent information about government buffer stockholdings in developing and lower-middle-income countries may impact private buffer stockholdings and what this could imply for smallholder farmers' income in these countries.

## Declarations

### Author contribution statement

E. Abokyi and K.F. Asiedu: Conceived and designed the experiments; Analyzed and interpreted the data; Contributed reagents, materials, analysis tools or data; Wrote the paper.

D. Strijker and M.N. Daams: Analyzed and interpreted the data; Contributed reagents, materials, analysis tools or data; Wrote the paper.

### Funding statement

This research did not receive any specific grant from funding agencies in the public, commercial, or not-for-profit sectors.

### Competing interest statement

The authors declare no conflict of interest.

### Additional information

No additional information is available for this paper.

## Figures and Tables

**Table A1:** Balance Checking Before and After Neighbour, Caliper, and Radius Matchings.

Covariates	Sample	Kernel			Nearest Neighbour					Caliper Matching				Radius Matching			
		Mean		t-test	Mean			t-test		Mean		t-test		Mean		t-test	
		Treated	Control	T	p>z	Treated	Control	T	p>z	Treated	Control	T	p>z	Treated	Control	T	p>z
*Gen*	Unmatched	0.78	0.67	2.82	0.01	0.78	0.67	2.82	0.01	0.784	0.673	2.82	0.01	0.78	0.67	2.82	0.01
	Matched	0.85	0.83	0.59	0.56	0.85	0.83	0.68	0.50	0.850	0.825	0.68	0.50	0.85	0.83	0.59	0.56
*Ln(Age)*	Unmatched	3.88	3.85	1.16	0.25	3.88	3.85	1.16	0.25	3.875	3.850	1.16	0.25	3.88	3.85	1.16	0.25
	Matched	3.90	3.89	0.24	0.81	3.90	3.89	0.19	0.85	3.90	3.89	0.19	0.85	3.90	3.88	0.70	0.49
*Mar*	Unmatched	0.76	0.80	-0.92	0.36	0.76	0.80	-0.92	0.36	0.76	0.80	-0.92	0.36	0.76	0.80	-0.92	0.36
	Matched	0.86	0.87	-0.30	0.77	0.86	0.84	0.42	0.68	0.86	0.84	0.42	0.68	0.86	0.86	-0.17	0.87
*Edu*	Unmatched	2.42	2.29	1.64	0.10	2.42	2.29	1.64	0.10	2.42	2.29	1.64	0.10	2.42	2.29	1.64	0.10
	Matched	2.43	2.36	0.81	0.42	2.43	2.42	0.13	0.90	2.43	2.42	0.13	0.90	2.43	2.41	0.21	0.84
*Market*	Unmatched	2.84	3.05	-3.23	0.00	2.84	3.05	-3.23	0.00	2.84	3.05	-3.23	0.00	2.84	3.05	-3.23	0.00
	Matched	2.84	2.98	-2.10	0.04	2.84	2.98	-2.20	0.03	2.84	2.98	-2.2	0.03	2.84	2.95	-1.74	0.08
*Ln(Pack)*	Unmatched	3.95	3.86	2.84	0.01	3.95	3.86	2.84	0.01	3.95	3.86	2.84	0.01	3.95	3.86	2.84	0.01
	Matched	3.95	3.95	-0.38	0.70	3.95	3.96	-0.92	0.36	3.95	3.96	-0.92	0.36	3.95	3.95	-0.25	0.80
*Ln(Trans)*	Unmatched	4.01	3.81	5.85	0.00	4.01	3.81	5.85	0.00	4.00	3.81	5.85	0.00	4.01	3.81	5.85	0.00
	Matched	4.01	4.01	0.02	0.98	4.01	4.02	-0.46	0.64	4.01	4.02	-0.46	0.64	4.01	4.01	0.08	0.93
*Ext*	Unmatched	4.27	3.82	11.68	0.00	4.27	3.82	11.68	0.00	4.27	3.82	11.68	0.00	4.27	3.82	11.68	0.00
	Matched	4.30	4.32	-0.65	0.52	4.30	4.30	-0.15	0.88	4.30	4.30	-0.15	0.88	4.30	4.34	-1.09	0.27
Ln (Exp)	Unmatched	3.33	3.23	1.59	0.11	3.33	3.23	1.59	0.11	3.33	3.23	1.59	0.11	3.33	3.23	1.59	0.11
	Matched	3.33	3.42	-1.39	0.16	3.33	3.57	-4.33	0.00	3.33	3.57	-4.33	0.00	3.33	3.47	-2.27	0.02
HS	Unmatched	5.58	5.90	-1.56	0.12	5.58	5.90	-1.56	0.12	5.58	5.90	-1.56	0.12	5.58	5.90	-1.56	0.12
	Matched	5.64	5.77	-0.64	0.52	5.64	5.64	0.00	1.00	5.64	5.64	0.00	1.00	5.64	5.77	-0.68	0.50

**Table A2:** Endogeneity Test.

Variable	Total farm income	Farm income per output
*NAFCO*	0.805∗∗∗ (0.084)	0.834∗∗∗(0.086)
*Ln(age)*	-0.621∗ (0.276)	-0.808∗∗∗ (0.283)
*Mar*	0.094 (0.188)	0.121 (0.192)
*Gen*	0.122 (0.254)	0.013 (0.260)
*Edu*	0.145∗ (0.080)	0.138∗ (0.082)
*HS*	0.090∗∗∗ (0.032)	-0.078∗∗ (0.033)
*Residual*	1.624 (1.319)	1.864 (1.351)
*Constant*	7.061∗∗∗ (0.729)	7.034∗∗∗ (0.747)
F-stat	18.30∗∗∗	23.76∗∗∗
R-square	0.253	0.306
RMSE	0.813	0.832
Obs.	386	386

Notes∗∗∗, ∗∗, ∗; are values statistically significant at 0.01, 0.05, and 0.1 levels, respectively. The standard errors are in parentheses. Note that the residual is not significant, indicating that endogeneity is not a problem.

## Footnotes

1 We use the term EU also for EEC (European Economic Community (EEC) and EC (European Communities)).

2 The weights are to compensate for the differential strata sizes during the matching. For example, if we denote $T^{s}$ as the treated units in stratum *s*, with count $m_{T}^{s}$ = # $T^{s}$ the number of treated units in the stratum. Similarly, for the control units, that is, $m_{C}^{s}$ = # $C^{s}\;$. The number of matched units are, respectively, for treated and controls, $m_{T}=\cup_{s\in S}m_{T}^{s}$ and $m_{C}=\cup_{s\in S}m_{C}^{s}$. To each matched unit *i* in stratum s, CEM assigns the weights (W_i_) as (Iacus et al., 2011): $w_{i}=\left\{ \begin{array}{cc} 1, & i\in T^{s}\\ \frac{m_{C}}{m_{T}}*\frac{m_{T}^{s}}{m_{C}^{s}}, & i\in C^{s} \end{array}\right.$, where the unmatched units receive weight w_*i*_*=0.*

3 The MD figure of 0 means the data between the treated and control is balanced in specific quantiles, 1 means the data is not balanced in those quantiles. Except for HH at min quantile, all other variables are balanced in all quantiles of the distributions.

4 The CEM process divided the data into a total of 100 strata with 44 being matched strata (Mishra and Sam, 2016).
